# Semiconducting Tungsten Trioxide Thin Films for High-Performance SERS Biosensors

**DOI:** 10.3390/nano15181393

**Published:** 2025-09-10

**Authors:** Hao Liu, Liping Chen, Bicheng Li, Haizeng Song, Chee Leong Tan, Yi Shi, Shancheng Yan

**Affiliations:** 1 School of Integrated Circuit Science and Engineering, Nanjing University of Posts and Telecommunications, Nanjing 210023, China; 2023221102@njupt.edu.cn (H.L.); cheelong@gmail.com (C.L.T.); 2 School of Chemistry and Life Sciences, Nanjing University of Posts and Telecommunications, Nanjing 210023, China; 1023173009@njupt.edu.cn; 3 Advanced Materials Laboratory, Fudan University, Shanghai 200438, China; 23113010019@m.fudan.edu.cn; 4 School of Electronic Science and Engineering, Nanjing University, Nanjing 210093, China; songhaizeng0501@foxmail.com; 5 Henan Key Laboratory of Rare Earth Functional Materials, Zhoukou Normal University, Zhoukou 466001, China

**Keywords:** surface-enhanced Raman scattering, tungsten trioxide, biosensors, molecular detection

## Abstract

Surface-enhanced Raman Scattering (SERS) enables ultrasensitive detection but is often hindered by biocompatibility and sustainability concerns due to its reliance on noble metal substrates. To overcome these limitations, we develop a semiconductor-based SERS platform utilizing ultrathin tungsten trioxide (WO_3_) nanofilms synthesized via a facile annealing process on fluorine-doped tin oxide (FTO). This system achieves an impressive Raman enhancement factor of 1.36 × 10^6^, enabling ultrasensitive detection of rhodamine 6G (R6G) and methylene blue (MB) at ultralow concentrations, surpassing conventional metal-based SERS platforms. It is further suggested that this is a substrate that can be easily coupled to other metals. An application for the detection of adenine molecules is realized through layered WO_3_-Au NPs composites, where embedded gold nanoparticles act as plasma “hot spots” to amplify the sensitivity. Density functional theory (DFT) calculations and band structure analysis confirm that synergistic interface charge transfer and naturally formed oxygen vacancies enhance performance. By combining semiconductor compatibility with other metal amplification, this WO_3_-based SERS platform offers a sustainable and high-performance alternative to conventional substrates, paving the way for environmentally friendly and scalable Raman sensing technologies.

## 1. Introduction

Surface-enhanced Raman spectroscopy (SERS) has emerged as a vital analytical tool, surpassing traditional trace detection methods with its ultra-high sensitivity (single-molecule detection) and molecular specificity. Originating from the observation of enhanced Raman signals from pyridine on rough silver electrodes [1], SERS has evolved from fundamental studies to diverse applications, including cancer biomarker detection, portable diagnostic devices, and environmental pollutant analysis [2,3,4]. Its advantages include label-free operation, high interference resistance, and in situ detection [5]. SERS enhancement relies on electromagnetic (EM) and chemical (CM) mechanisms, with recent advances exploring novel strategies based on these principles [6,7].

While noble metals (e.g., Au, Ag) dominate clinical SERS due to localized surface plasmon resonance (LSPR) effects [8], their high cost and biocompatibility limitations have spurred interest in non-noble semiconductor substrates [9,10]. The materials like MoO_3−x_ quantum dots, Ti_3_C_2_T_x_ nanosheets, Fe_3_O_4_ crystals, and MXene/MoS_2_ hybrids demonstrate tunable SERS performance through bandgap engineering, oxygen vacancy introduction, or nanostructure optimization [11,12,13]. WO_3_ has gained attention since discovered its interfacial enhancement with Ag [14]. Subsequent studies on WO_3_ nanosheets, hollow spheres, and oxygen-deficient WO_3−x_ nanorods have expanded their applications in catalysis, biomedicine, and sensors [15,16,17].

Despite progress, challenges persist in substrate synthesis complexity, reproducibility, and material defects. Innovations like ZnO-based 3D quantum probes [18] and Fe_3_O_4_@Au magnetic SERS-LFA systems [19] highlight efforts to improve practicality. Sakir et al. developed a reusable, photocatalytic SERS substrate using Au/ZnO on a membrane, achieving a detection limit of 10^−10^ M for R6G [20]. Korkmaz et al. created a superhydrophobic Ag@ZnO@Bi_2_WO_6_ SERS substrate, reporting an enhancement factor of 1.2 × 10^5^ for R6G [21]. Shavlaya et al. fabricated a reusable Au/Pd@Cu_2_O sensor with an enhancement factor of 5 × 10^5^ for R6G, which could detect it at 10^−6^ M and was cleaned via oxygen plasma treatment [22]. Current research focuses on developing cost-effective, biosafe substrates with simplified fabrication [23,24]. This work proposes a reproducible strategy for ultrathin WO_3_ nanofilms, balancing high SERS performance, structural control, and detection accuracy while addressing scalability challenges in real-world applications [25].

This study synthesized a WO_3_ nanofilm with homogeneity and stability as a highly sensitive surface Raman-enhanced substrate. We deposited ultrathin 3 nm WO_3_ nanofilm on a conductive glass FTO using a simple Sol–Gel and annealing method. This SERS substrate achieved detection limits of 1 × 10^−9^ M and 1 × 10^−8^ M for R6G and methylene blue, respectively, with the highest enhancement factor of 1.36 × 10^6^. Synthesis does not require a complex defect-tuning process with narrow band gaps and surface defects. These changes occur naturally during self-aggregation, causing an increase in sub-bandgap states and free electrons, which may facilitate resonance coupling and CT processes. Adenine, as a component of DNA and RNA, plays a key role in transmitting and expressing genetic information. We synthesized Au particle “hot spot” modified WO_3_ nanosensors (WO_3_-Au NPs) instead of the traditional noble metal by interfacial mosaicing method. We realized a low detection limit of 1 × 10^−10^ M for adenine molecules. This paper provides an easy-to-prepare and straightforward new method for designing new sensitive biomolecule-detecting SERS substrates.

## 2. Materials and Methods

### 2.1. Materials and Chemicals

Tungstic Acid (H_2_WO_4_, ≥99%), Nitric Acid (HNO_3_, 70%), and Hydrogen Peroxide (H_2_O_2_, 30%) Tetrachloroalloyed acid (HAuCl_4_, Au 23.5~23.8% in dilute HCl), Cetyltrimethylammonium chloride (CTAC, ≥97%), Sodium tetrahydroborate (NaBH_4_, 2.0 M in triethylene glycol dimethyl ether), Potassium iodide (KI, ≥99%), Ascorbic acid (AA, ≥97%), Cetyltrimethylammonium bromide (CTAB, ≥99%) were purchased from Aladdin Reagent (Shanghai) Co., Ltd., Shanghai, China. Rhodamine 6G (R6G), Methylene Blue (MB) and Adenine were purchased from Sigma-Aldrich (Shanghai) Trading Co., Ltd., Shanghai, China.

### 2.2. Materials Characterization

Transmission Electron Microscopy (TEM), High Resolution Transmission Electron Microscope (HRTEM), and Selected Area Electron Diffraction (SAED) are used by Tecnai G2 F30 300KV instrument (Yangzhou University, manufactured by FEI Company, Shanghai, China); Scanning Electron Microscopy (SEM) and Energy Dispersive Spectroscopy (EDS) are used by JEOL JSM-IT800 (Zhoukou Normal University, manufactured by JEOL (Beijing) Sci-Tech Co., Ltd., Beijing, China); Ultraviolet-Visible Spectrophotometer (UV-vis spectrophotometer) is used by PERSEE TU 1810 (Nanjing University of Posts and Telecommunications, manufactured by PERSEE Company, Beijing, China); Raman spectra is used by the Mstarter 100 Microspectral Scanning Test System (Nanjing University of Posts and Telecommunications, manufactured by Nanjing Metatest Optoelectronic Technology Co., Ltd., Nanjing, China).

### 2.3. Preparation of Precursor Gels and Ultrathin WO_3_ Nanofilm

**Figure 1:**
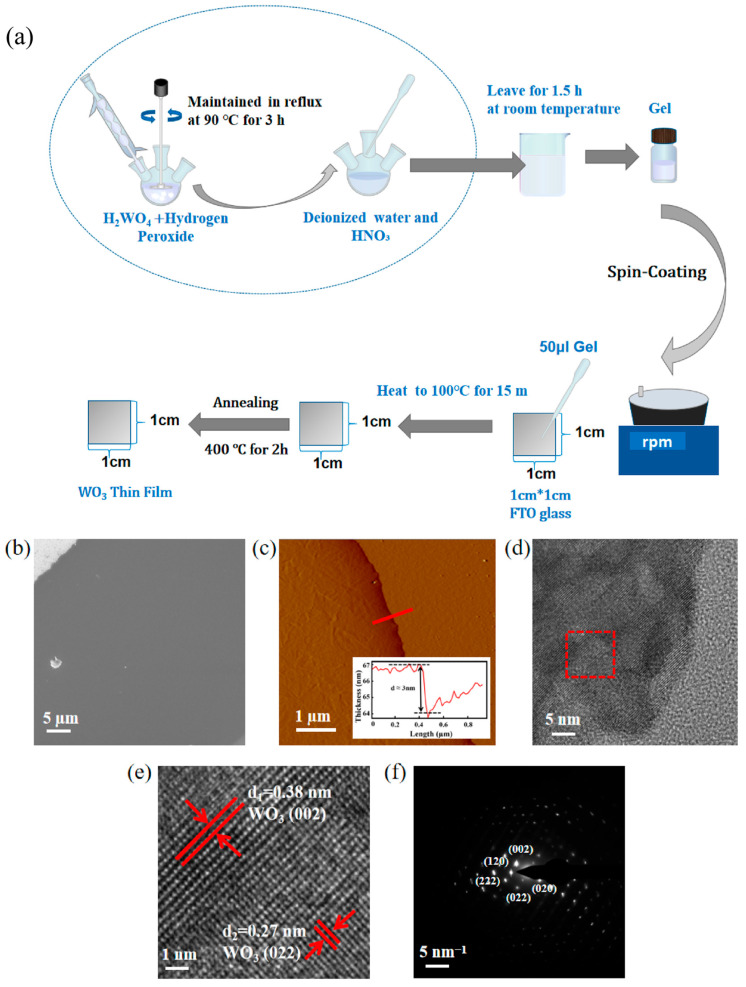
(**a**) Flow of synthesizing WO_3_ nanofilm. (**b**) SEM image of WO_3_ thin film. (**c**) AFM image of WO_3_ nanofilm and height profile of the red line section (inset). (**d**) TEM image of WO_3_ nanofilm. (**e**) HRTEM image of WO_3_ nanofilm (Captured in the red-boxed area in Figure d, the red arrows point to different crystal planes). (**f**) SAED image of WO_3_ nanofilm.

1.0 g of H_2_WO_4_ was mixed with 20 mL of 30% H_2_O_2_ and refluxed at 90 °C for 3 h to produce a solution of clarified yellow tungsten peroxo complex. After dilution to 0.3 M, nitric acid was added dropwise to adjust the pH to 2–3 and filtered. The FTO substrate was cleaned sequentially by acetone, isopropanol, deionised water by ultrasonication, and blown dry by nitrogen. 50 μL of the solution was spin-coated onto the substrate: the first section was spread evenly at 1500 rpm/10 s, and the second section was densified at 3500 rpm/30–60 s [26]. The membrane was heated at 100 °C for 15 min. The wet film was heated at 100 °C for 15 min to remove residual solvents and peroxides, followed by vacuum-programmed heating to 400 °C for 2 h. Finally, WO_3_ semiconducting thin films with a thickness of 3 nm were fabricated. The synthesis flow is shown in Figure 1a.

### 2.4. Measurement of SERS Performance

All SERS samples were prepared using the conventional immersion method. Before the SERS assay, film samples were, respectively, stored in R6G solution and MB solution of different concentrations (10^−5^–10^−9^ M, solvent: water.) for 60 min, then removed and rinsed thoroughly. The cumulative exposure time was 10 s, and the laser power density was about 0.7 mW·cm^−2^. For blank samples, only 10 μL of R6G or MB solutions were dropped on a clean FTO and dried at 60 °C. Raman spectra were acquired using a Raman spectrometer with a 532 nm laser. The spot diameter was 1.5 μm. The exposure time was 10 s.

### 2.5. Preparation of Adenine Biosensors and SERS Measurements

HAuCl_4_ (100 µL, 25 mmol/L), CTAC (5 mL, 0.2 mol/L) and H_2_O (4.9 mL) were mixed, and pre-cooled NaBH_4_ (450 µL, 20 mmol/L) was added to obtain light brown gold seed solution. After maturation, growth matrix solutions containing CTAC, HAuCl_4_, KI and AA were injected separately to promote crystal nucleus development. After centrifugation, they resuspended in CTAB. After washing, the gold seeds were added to the growth solution containing CTAB, HAuCl_4_ and AA. 20 µL of gold seeds were introduced and gently stirred. The gold nanoparticles were incubated at a constant temperature of 30 °C and left to stand for 2 h. Then, 20 μL of the prepared solution containing a certain number of Au nanoparticles was uniformly deposited on the FTO-WO_3_ film (5–15 particles/μm^2^), which was put into a vacuum drying oven at 60 °C for the modification treatment, and the semiconductor film containing Au particles was taken out. The semiconductor sensors were kept in different concentrations of adenine molecule solutions (10^−6^–10^−10^ M, solvent: water at 60 °C) for 60 min, removed, and thoroughly cleaned, and specific Raman signals of the target molecules were measured. The detection of adenine was completed against a blank group based on the difference.

### 2.6. Calculation of the R6G Raman Enhancement Factor (EF)

The Raman coefficients of the WO_3_ SERS substrate were calculated according to the following formula:

(1) $$EF=\frac{I_{SERS}}{I_{bulk}}\cdot \frac{N_{bulk}}{N_{SERS}}$$

In SERS quantification, I_SERS_ represents the Raman signal intensity measured under surface-enhanced conditions, while N_SERS_ represents the number of molecules interacting with the sample within the SERS-active region. Correspondingly, I_bulk_ refers to the Raman signal intensity acquired under non-enhanced conditions, and N_bulk_ refers to the number of molecules interacting with the sample in the absence of enhancement. [27]. For the experiments, the excitation wavelength was 532 nm, the laser power was 0.7 mW, and the integration time was 10 s. The EFs were estimated using the R6G peak at P1 or P2. Specific calculations can be found in the Supporting Information.

## 3. Results and Discussion

### 3.1. Synthesis and Characterization

A synthesis strategy is introduced here that eliminates the need for templates and acids. It avoids traditional methods for preparing tungsten oxide thin films, which often require complex templates or harsh acid treatments. The SEM images of the synthesized WO_3_ nanofilm at the edges are shown in Figure 1b. WO_3_ nanofilms have a uniform and crack-free surface topography with good interfacial contact with the FTO substrate. This is the key to realize a high specific surface area and provide abundant active sites for SERS. The AFM images of the film edges are shown in Figure 1c and further validate the ultrathin nature of the films with a uniform thickness distribution (~3 nm) and a more uniform and consistent surface roughness (Supplementary Figure S1). The maximum height difference in the surface within the area is about 2.791 nm, and the root mean square roughness (RMS) is about 0.13 nm. To verify the physical phase and lattice arrangement of the WO_3_ nanofilms. Figure 1d demonstrates the TEM image of the WO_3_ nanofilms, in which the HRTEM image of the red-boxed portion is shown in Figure 1e, which shows that the nanofilms have a single-crystal property. The HRTEM image reveals distinct lattice spacings of 0.38 nm and 0.27 nm, matching the (002) and (022) planes of monoclinic WO_3_ (JCPDS 43-1035). Among them, the (002) crystal surface corresponds to the monoclinic phase, (022) crystal surface corresponds to the high index face, which confirms that the crystals are naturally produced with oxygen vacancy defects. Figure 1f displays the SAED pattern of the nanofilm, showing a lattice alignment along the crystallographic zone axis.

**Figure 2:**
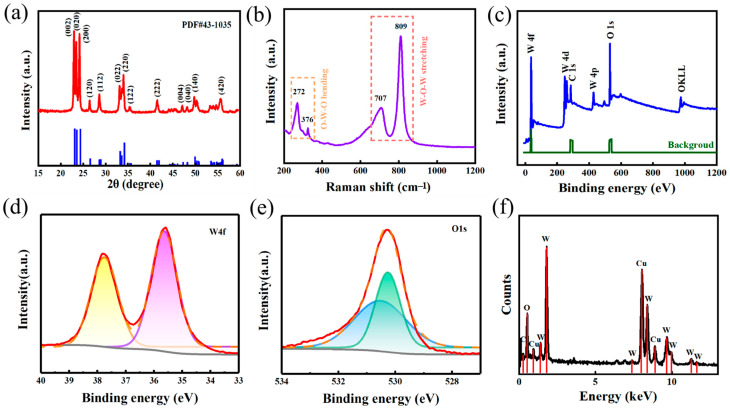
(**a**) XRD image of tungsten trioxide. (**b**) Raman spectral image of tungsten trioxide. (**c**) XPS full-spectrum image of WO_3_ nanofilm. (**d**) W4f spectral image of WO_3_ nanofilm. (**e**) O1s spectral image of WO_3_ nanofilm. (**f**) EDS images of WO_3_ nanofilm.

Figure 2a shows the XRD spectrum of the WO_3_ film. All diffraction peaks exhibit strict congruence with the reference monoclinic WO_3_ phase (JCPDS 43-1035), particularly matching dominant lattice planes such as (002), (020), and (200). In Figure 2b, Raman spectroscopic analysis reveals distinct vibrational modes characteristics of monoclinic WO_3_ symmetry. Sharp peaks indicate a highly ordered WO_3_ crystal arrangement. Peaks at 272 cm^−1^ and 376 cm^−1^ are attributed to O-W-O bending vibrations, while the strong peaks at 707 cm^−1^ and 809 cm^−1^ arise from W-O-W stretching vibrations. The absence of stray peaks confirms uniform chemical bonding.

XPS analysis of WO_3_ nanofilms (Figure 2c) confirms W and O as primary components, with W4p, W4d, W4f, and O1s peaks verifying structural integrity. The W4f spectrum (Figure 2d) exhibits characteristic W^6+^ doublet peaks at 35.5 eV (W4f 5/2) and 37.7 eV (W4f7/2). O1s analysis (Figure 2e) reveals lattice oxygen (530.27 eV) and surface-adsorbed oxygen (530.55 eV). Binding energy shifts [28] indicate reduced electron density at oxygen sites, correlating with defect formation. These defects modify the material’s surface electronic structure and establish interfacial transport channels for dye molecule electron transfer. Figure 2f is the distribution of complementary EDS elements showing no impurity peaks, which contains Cu for testing as well as W and O elements, indicating the chemical integrity of the synthesized nanomembranes. The above structural advantages synergize with excellent crystallinity and surface oxygen defects, which are essential for the application of surface-enhanced Raman scattering.

### 3.2. SERS Properties and Enhancement Mechanism of Ultrathin WO_3_ Nanofilm

**Figure 3:**
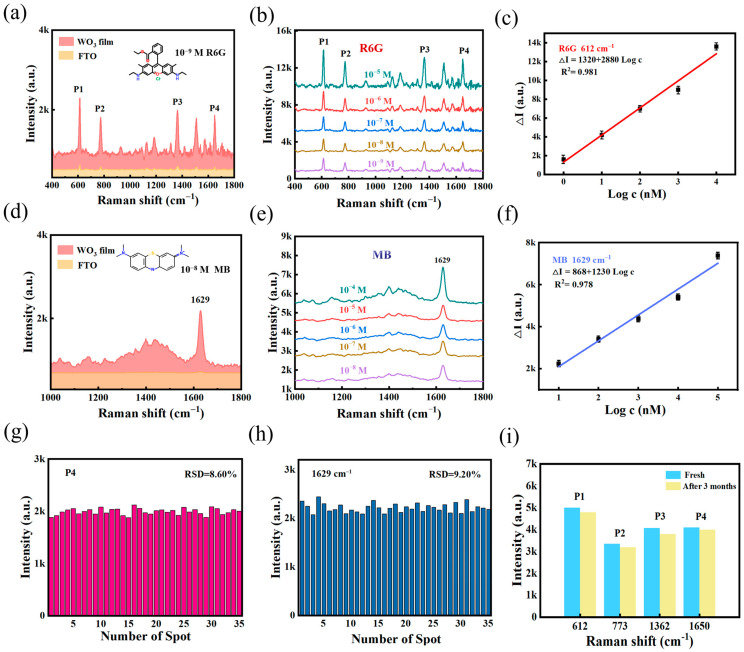
(**a**) Raman spectra of R6G on substrates. (**b**) Comparison of the detection of different concentrations of R6G by WO_3_ nanofilm. (**c**) Calibration plot of SERS intensity versus the logarithm of concentration for detecting R6G molecule at 612 cm^−1^. (**d**) Raman spectra of MB on substrates. (**e**) Comparison of the detection of different concentrations of MB by WO_3_ nanofilm. (**f**) Calibration plot of SERS intensity versus the logarithm of concentration for detecting MB molecule at 1629 cm^−1^. (**g**) RSD value for detecting R6G (10^−9^ M) with a peak at 1650 cm^−1^. (**h**) RSD value for detecting MB (10^−8^ M) with a peak at 1629 cm^−1^. (**i**) Three-month time comparison of the WO_3_ nanofilm for R6G.

R6G and MB were employed as Raman probes to evaluate the SERS performance of WO_3_ film. Figure 3a shows the SERS spectra of adsorbed R6G molecules with and without substrate. Four distinct characteristic peaks of R6G (P1 612 cm^−1^, P2 773 cm^−1^, P3 1362 cm^−1^, P4 1650 cm^−1^) were detected by the substrate when the R6G concentration varied from 10^−5^ to 10^−9^ M. Within the 1 nM^−10^ μM range, Figure 3b,c revealed a linear correlation of P1(ΔI = 1320 + 2880 log C (nM), yielding an R2 value of 0.981. Similarly, the calibration plots of SERS intensity versus logarithm of concentration for the detection of R6G molecules at P2, P3, P4 are shown in Supplementary Figure S2. Notably, the responses of peaks P1 and P2 are higher than those of P3 and P4. According to the Herzberg-Teller selection rule [29], the specific vibrational modes of the adsorbed molecules (e.g., non-completely symmetric modes) undergo selective signal amplification when charge transfer dominates Raman enhancement. The asymmetric vibrational modes of R6G adsorbed on the semiconductor surface show significant enhancement compared to the symmetric modes, confirming that CT within this system is the main mechanism driving Raman enhancement.

The capture of MB molecules is shown in Figure 3d. The limit of detection (LOD) is another important factor for SERS. The LOD of R6G and MB from WO_3_ substrate could reach 10^−9^ (Figure 3b) and 10^−8^ M (Figure 3e), respectively. Figure 3f demonstrates the linear response at a peak of 1629 cm^−1^ (ΔI = 868 + 1230 log C (nM), R2 = 0.978). To quantitatively estimate the SERS effect, the EF can be calculated using formulas (1). As seen in Supplementary Figure S3, the highest EF of 1.36×10^6^ was achieved using the R6G molecule as a probe, a high performance in non-noble metal substrates (Supplementary Table S1).

Critical factors in the evaluation of SERS substrates include homogeneity, sensitivity and stability. The SERS intensity variations in R6G and MB at 35 random positions on the WO_3_ membrane were analyzed separately (Figure 3g,h), resulting in relative standard deviations (RSDs) of 8.60% and 9.20%, respectively. Supplementary Figure S4 shows the Raman mapping images for R6G and MB, revealing consistent intensities. Stability tests (Figure 3i) showed negligible peak shifts or intensity degradation over a period of three months and retained the ability to enhance the four prominent peaks. These results validate the long-term stability and practical applicability of the substrate.

**Figure 4:**
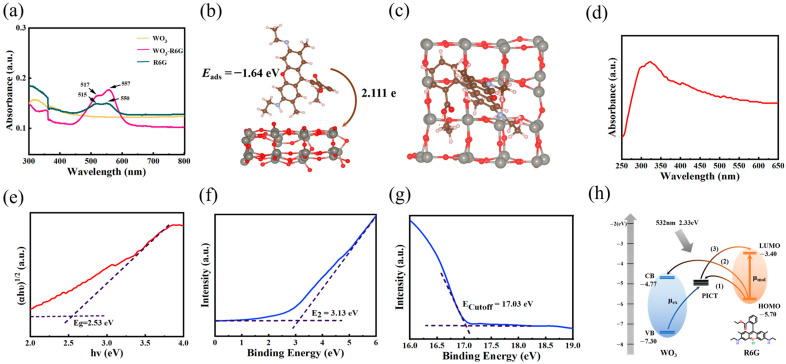
Mechanism analysis of the SERS substrate. (**a**) UV-vis absorption spectra of WO_3_, R6G-modified WO_3_ film, and R6G solution. (**b**) DFT calculations of R6G molecules adsorbed on WO_3_ (Side view). (**c**) DFT calculations of R6G molecules adsorbed on WO_3_ (Top view). (**d**) UV-vis absorption spectra of WO_3_ nanofilm. (**e**) The corresponding (αhν)^1/2^ versus hν correspondence curves. (**f**,**g**) UPS spectra of WO_3_ nanofilm. (**h**) The analysis of CT path in WO_3_/R6G.

CM specifically explored the enhancement mechanism of WO_3_ substrates [30]. The UV-VIS absorption spectra of WO_3_, R6G-modified WO_3_ film, and R6G solution were measured (Figure 4a). New absorption peaks were found near 517 and 557 nm, and a certain degree of peak broadening was observed in the composite absorption spectra, indicating the existence of charge transfer (CT) processes and chemical interactions between the substrate and the molecule under 532 nm excitation. This performance may be attributed to synergistic coupling between charge redistribution mechanisms and defect energy levels [31].

To elucidate the variation in the electron density in the SER, the adsorption structure and CT behavior between R6G and WO_3_ were investigated by density functional theory (DFT) (Figure 4b,c). The adsorption energy of the WO_3_/R6G system is −1.64 eV, suggesting a stronger coupling between the two. In addition, CT analysis using the BADER atomic quantum theory shows an increase in charge on WO_3_ and a molecular charge transfer of 2.111 e to the substrate. The stronger adsorption energy and more efficient CT process show better CM performance compared to other WO_3_ systems without natural oxygen vacancies [32].

Figure 4d,e show the plots of the UV-vis spectra and (αhν)^1/2^ against hν, respectively. According to the research of J. Tauc et al., [33], the band gap of the WO_3_ nanomaterials is about 2.53 eV. From the UPS spectra of WO_3_ nanofilm (Figure 4f,g), information about the bandgap of the current substrate is obtained, including the valence band (VB) and the conduction band (CB) of WO_3_. Based on Formulas (2)–(4), we derive the ultraviolet photoelectron spectroscopy (UPS) of WO3 with a figure of merit of 4.17 eV, with the VB located at 7.3 eV and the CB at 4.77 eV.

(2) $$W_{F}=h\nu -E_{cutoff}$$

(3) $$VB=W_{F}+E_{2}$$

(4) $$CB=VB-E_{g}$$

Here, W_F_: Work function; h: Planck constant; ν: Frequency of the incident photon; E_cutoff_: Cutoff energy; VB: Valence Band maximum; E_2_: A specific energy parameter; CB: Conduction Band minimum; E_g_: Band gap energy.

Figure 4h illustrates the Photo-Induced Charge Transfer (PICT) process between the R6G molecule and WO_3_ nanofilm under the excitation of the incident photon (2.33 eV). Therein, the R6G molecule exhibits specific electronic energy level features: its lowest unoccupied molecular orbital (LUMO) is located at the −3.4 eV energy level, while the highest occupied molecular orbital (HOMO) is at the −5.70 eV energy level.

Notably, when R6G molecules are adsorbed on the surface of the substrate, it can promote the formation of a strong electronic coupling system between the adsorbed molecules and the substrate. This energy level arrangement not only enhances the tunnelling effect of the surface electrons, but also significantly improves the charge transfer efficiency at the interface, thus providing an optimized path for the surface-enhanced Raman scattering effect. These two strong resonances can be vibrationally coupled to give intensity to nearby PICT resonances. According to the energy matching principle, thermodynamically feasible PICT resonances may be related to the overall CM enhancement in our WO_3_-R6G system, including a total of (1) PICT between the molecular ground state and the oxygen vacancy-associated electronic state, (2) PICT between the molecular ground state and the CB state, and (3) PICT between the oxygen vacancy-associated electronic state and the molecular excited state.

### 3.3. Biosensors for the Detection of Adenine

As a core component of DNA/RNA bases and energy molecules (ATP), adenine is key in genetic information transfer and energy metabolism. Thus, a gold particle mosaic modeled WO_3_-Au NPs composite SERS sensor is designed to detect adenine molecules.

**Figure 5:**
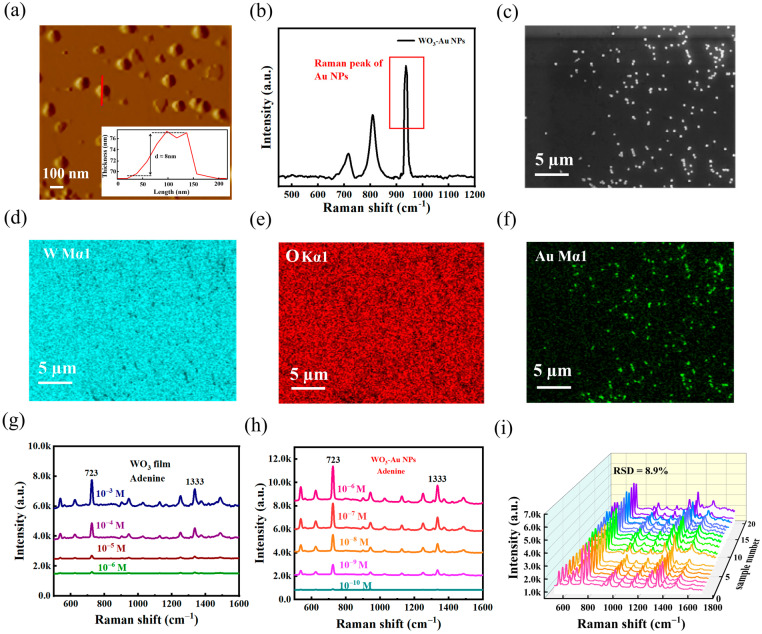
(**a**) AFM image of WO_3_-Au NPs. (**b**) Raman spectrum of WO_3_-Au NPs; (**c**) SEM image of WO_3_-Au NPs. (**d**) EDS Mapping image of W element. (**e**) EDS Mapping image of O element, and (**f**) EDS Mapping image of Au element. (**g**) Comparison of the detection of adenine molecules at different concentrations by WO_3_ nanofilm. (**h**) Comparison of the detection of adenine molecules at different concentrations by WO_3_-Au NPs. (**i**) Raman spectra of SERS of adenine (10^−9^ M) were collected at 20 random sites on WO_3_-Au NPs.

Figure 5a shows the AFM image of the “hotspot” of Au particles mosaiced on the film surface, the Au particles in the WO_3_-Au NPs are uniformly distributed and of uniform size, forming a stable and relatively dense “hotspot” pattern based on the film. The electromagnetic enhancement and chemical enhancement work together to form a large enhancement region [34]. Figure 5b shows the Raman spectra of WO_3_-Au NPs. Among them, the sharp broad peaks at 930–950 cm^−1^ are the characteristic peaks of Au particles, and the clear characteristic peaks reflect that the composite structure of WO_3_-Au NPs is well stabilized.

Figure 5c is the SEM image of the WO_3_-Au NPs under a large area, and the SEM image shows that this composite model is relatively homogeneous. Figure 5d–f show the distribution of W, O, and Au elements in the WO_3_-Au NPs, respectively. The W and O elemental signals are continuously distributed in the two-dimensional plane without localized enrichment or depletion regions. The characteristic peaks in Supplementary Figure S5 are separated without peak shift or overlap, and the elemental ratio of W and O is higher than 1:3 in Supplementary Table S2, which indicates that the prepared WO_3_ films are rich in oxygen vacancies. The surface coverage density follows the design value of about 5–15 particles/μm^2^. This distribution pattern can effectively enhance the plasmon resonance effect on the substrate surface.

Figure 5g,h compare the adenine detection performance of WO_3_-Au NPs and pure WO_3_ film. While WO_3_ film detect adenine at 10^−6^ M, lower concentrations yield unreliable signals. In contrast, WO_3_-Au NPs exhibit a 10^4^-fold enhancement, with a broader detection range and improved limit of detection. Supplementary Figure S6 demonstrates the linear correlation (723 cm^−1^ and 1333 cm^−1^) between SERS intensity and adenine concentration. Reproducibility tests (Figure 5i) reveal an 8.9% relative standard deviation (RSD) (723 cm^−1^, 20 measurement points), confirming excellent stability. These results highlight the excellent SERS properties of WO_3_-Au NPs, enabling sensitive adenine detection at LODs of 10^−10^ M.

To illustrate the contributions of EM and CM to this system, Supplementary Figure S7 compares the detection ability and EFs of adenine molecules for WO_3_ thin film Au NPs, and WO_3_-Au NPs systems, respectively. The excellent performance brought by the WO_3_-Au NPs system is the result of the coupling of EM and CM. Supplementary Figure S8 further demonstrates the compatibility of WO_3_-Au NPs with three different substrates (SiO_2_/FTO/CaF_2_). The composite substrates still maintain high sensitivity on different deposition platforms.

## 4. Conclusions

This work presents a simple, template-free synthesis of an ultrathin 3 nm tungsten trioxide nanofilm, achieving superior SERS performance through a strong charge transfer mechanism. Our method is faster and more efficient than conventional approaches, offering a scalable pathway for high-sensitivity Raman detection.

Experiments demonstrate the substrate has an EF of 1.36 × 10^6^, with detection limits as low as 10^−9^ M for R6G and 10^−8^ M for MB—surpassing some noble-metal-based SERS substrates. Based on DFT calculations and band analysis, the remarkable Raman enhancement is attributed to increased oxygen vacancies, strong exciton resonance, and an elevated electronic DOS near the Fermi level, all contributing to an efficient PICT resonance. This effect facilitates enhanced charge escape, transfer pathways, and robust vibrational coupling between analytes and the SERS substrate. Furthermore, the WO_3_-Au NPs composite introduces a doping strategy that enhances Raman sensitivity, achieving an ultralow LOD of 10^−10^ M for adenine. Incorporating gold nanoparticles creates localized plasmonic “hot spots”, significantly improving molecular binding and enabling biosensing applications. Meanwhile, the composite strategy achieves ultra-high sensitivity biomolecule detection using a small amount of noble metals, and more importantly than the cost savings, a compatible SERS thin-film substrate is proposed.

This work establishes a new paradigm for semiconductor-based SERS chips, demonstrating how morphology control and hybrid composites can synergistically optimize Raman performance. These findings provide critical insights for developing next-generation biosensors, biomolecular diagnostics, and virus detection technologies, paving the way for sustainable, high-performance SERS platforms in real-world applications.

## Supplementary Materials

The following supporting information can be downloaded at: https://www.mdpi.com/article/10.3390/nano15181393/s1, Figure S1: AFM image of the center surface of WO_3_ nanofilm; Figure S2: (a) Calibration plot of SERS intensity versus the logarithm of concentration for detecting R6G molecule at 773 cm^−1^, respectively. (b) Calibration plot of SERS intensity versus the logarithm of concentration for detecting R6G molecule at 1362 cm^−1^, respectively. (c) Calibration plot of SERS intensity versus the logarithm of concentration for detecting R6G molecule at 1650 cm^−1^, respectively; Figure S3: Raman EF of P1 and P2 at different concentrations. Error lines are based on the standard deviation of 10 measurements at each concentration; Figure S4: Raman mapping images of R6G and MB in the SERS Measurement of WO_3_ films; Table S1: A comparison of the performance of this WO_3_ substrate with other non-precious metal substrates; Figure S5: General Elemental Distribution of WO_3_-Au NPs in EDS; Table S2: Ratio of O, W, and Au elements in WO_3_-Au NPs; Figure S6: Calibration plot of SERS intensity versus logarithm of concentration at 723 cm^−1^ and 1333 cm^−1^ for adenine; Figure S7: Raman spectra (average EFs) of WO_3_-Au NPs, WO_3_ and Au NPs; Figure S8: (a) Raman spectra of adenine molecules at 10^−5^ M concentration detected by FTO/FTO-WO_3_ film/FTO-WO_3_-Au NPs. (b) Raman spectra of adenine molecules at 10^−5^ M concentration detected by SiO_2_/SiO_2_-WO_3_ film/SiO_2_-WO_3_-Au NPs. (c) Raman spectra of adenine molecules at 10^−5^ M concentration detected by CaF_2_/CaF_2_-WO_3_ film/CaF_2_-WO_3_-Au NPs.
