# Supplementary material for: Semiconducting Tungsten Trioxide Thin Films for High-Performance SERS Biosensors
Source: Nanomaterials (Basel). 2025 Sep 10;15(18):1393. doi: 10.3390/nano15181393 (PMC12472386; doi:10.3390/nano15181393)
Supplement: Supplementary file 1 [file nanomaterials-15-01393-s001.zip › nanomaterials-3855854-supplementary.pdf]

Supplementary Information:

Semiconducting Tungsten Trioxide Thin Films for High-Performance SERS Biosensors

Hao Liu [1] , Liping Chen [2] , Bicheng Li [3] , Haizeng Song [4] , Chee Leong Tan [1] , *, Yi Shi [5] , *, Shancheng Yan [1] and *

[1] School of Integrated Circuit Science and Engineering, Nanjing University of Posts and Telecommunications, Nanjing 210023, China
[2] School of Chemistry and Life Sciences, Nanjing University of Posts and Telecommunications, Nanjing 210023, China
[3] Advanced Materials Laboratory, Fudan University, Shanghai 200438, China
[4] School of Electronic Science and Engineering, Nanjing University, 210093, Nanjing, China
[5] Henan Key Laboratory of Rare Earth Functional Materials, Zhoukou Normal University, Zhoukou 466001, China
* yansc@njupt.edu.cn

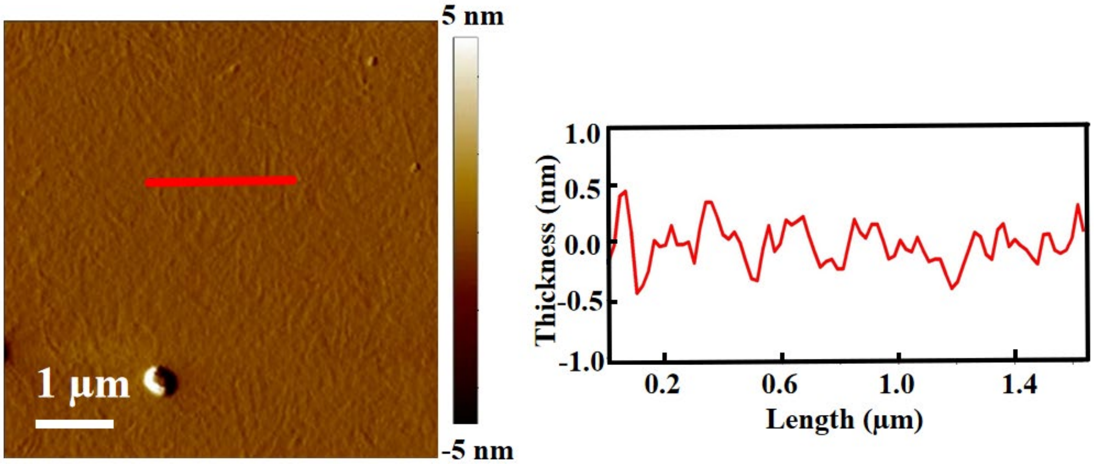

Supplementary Figure S1. AFM image of the center surface of WO_3 nanofilm.

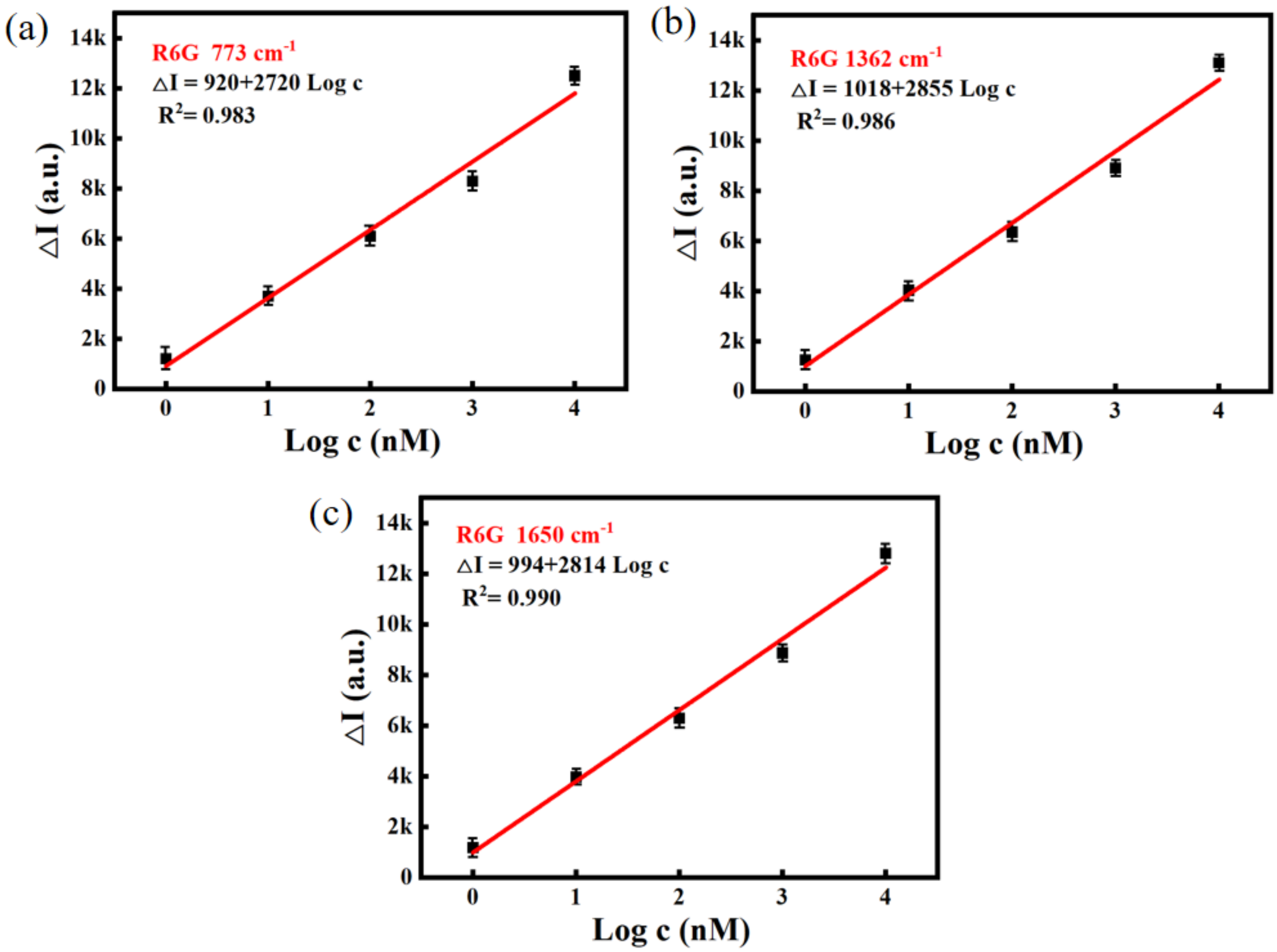

Supplementary Figure S2. (a) Calibration plot of SERS intensity versus the logarithm of concentration for detecting R6G molecule at 773 cm^{-1}, respectively. (b) Calibration plot of SERS intensity versus the logarithm of concentration for detecting R6G molecule at 1362 cm^{-1}, respectively. (c) Calibration plot of SERS intensity versus the logarithm of concentration for detecting R6G molecule at 1650 cm^{-1}, respectively.

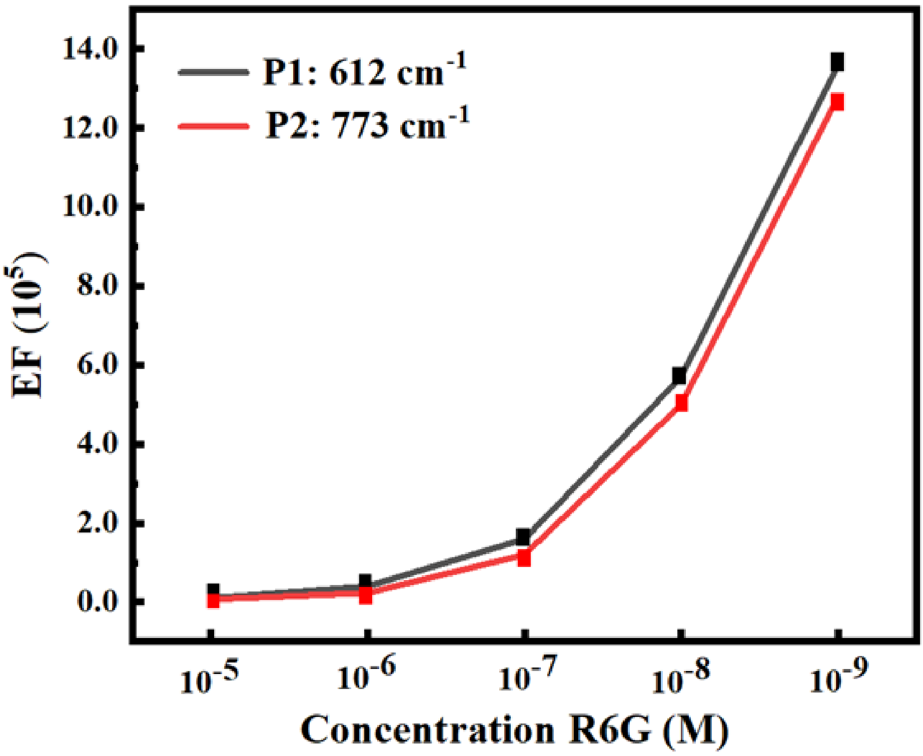

Supplementary Figure S3. Raman EF of P1 and P2 at different concentrations. Error lines are based on the standard deviation of 10 measurements at each concentration.

According to the following formula:

$$EF = \frac{I_{SERS}}{I_{bulk}}\frac{N_{bulk}}{N_{SERS}} \quad (1)$$

We obtained the average of the peaks (P1, P2) measured for 10 random samples as the calculated values for SERS measurements.

$$N_{SERS} = cvN_A \ (A_{Raman}/A_{Sub}) \quad (5)$$

$$N_{bulk} = dhMA_{Raman}N_A \quad (6)$$

where c represents the molar concentration of the analyte solution (mol/L), v is the volume of the analyte droplet, N_A is Avogadro's constant, and A_{Raman} is the area of the laser spot (1.5 μm in diameter). Based on the droplet area, A_{Sub} is about 0.5 cm^2, the laser penetration depth h is 21 μm, the molecular weight M=479.01 g/mol of R6G, and the density d is 1.15 g/cm^3. Therefore, N_{SERS} and N_{bulk} can be calculated using the formula (5) (6). The calculated EF of the R6G (10^{-9} M) is about 1.36×10^6.

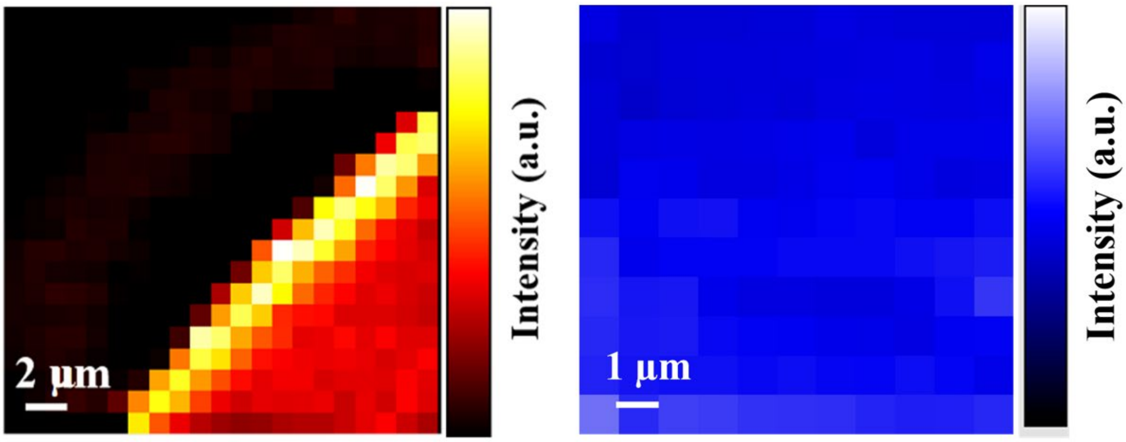

Supplementary Figure S4. Raman mapping images of R6G and MB in the SERS Measurement of WO_3 films.

Supplementary Table S1. A comparison of the performance of this WO_3 substrate with other non-precious metal substrates.

SERS Substrates	Target	EF	LOD	Reference
Au nanoparticles on ZnO nanorods	R6G; erythrosine B dyes	/	10^{-9} M;10^{-8} M	[20]
Ag@ZnO@Bi_2WO_6	RhB; R6G	3.2×10^5; 1.2×10^5	100 μM to 1 nM	[21]
Au/Pd@Cu_2O substrate	R6G	5×10^5	10^{-6}M	[22]
porous Tungsten Trioxide films	MB; vascular endothelial growth factor (VEGF)	1.5×10^6	8.7 pg/mL	[23]
$WC_{0.82}$ chip	R6G	2.31×10^5	/	[24]
WO_3 nanofilm; WO_3-Au NPs	R6G; Adenine	1.36×10^6; 3.50×10^7	10^{-9} M; 10^{-10} M	*This work

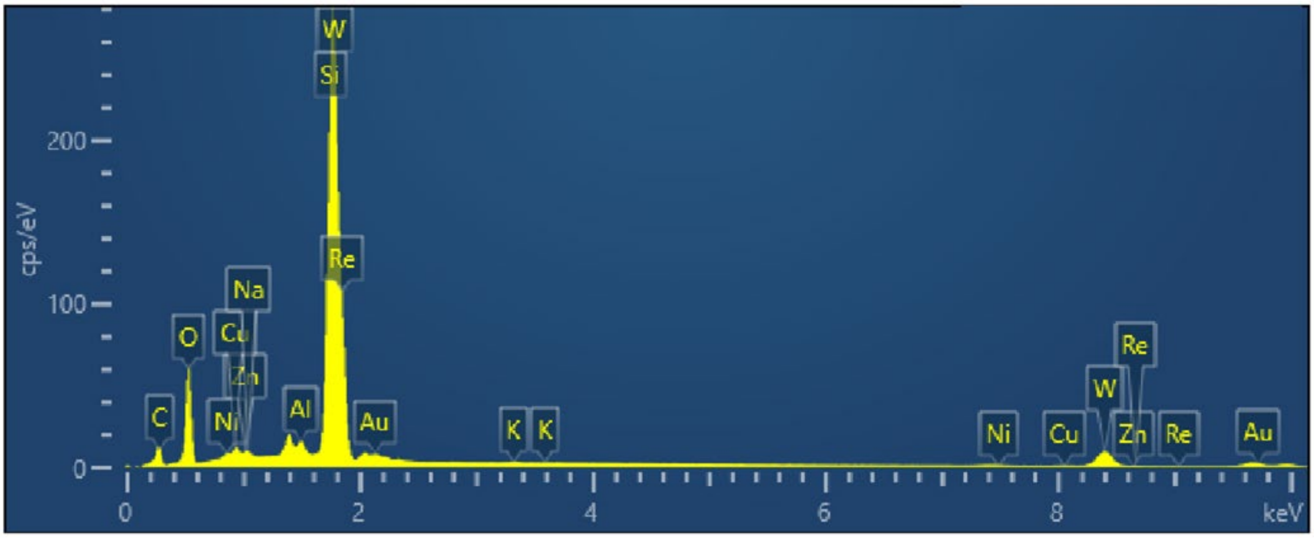

Supplementary Figure S5. General Elemental Distribution of WO_3-Au NPs in EDS.

Supplementary Table S2. Ratio of O, W, and Au elements in WO_3-Au NPs.

	**Distribution of major elements in WO_3-Au NPs**	
**Element**	**Wt%**	**Wt% Sigma**
**O**	21.91%	0.08
**W**	75.38%	0.16
**Au**	2.71%	0.10

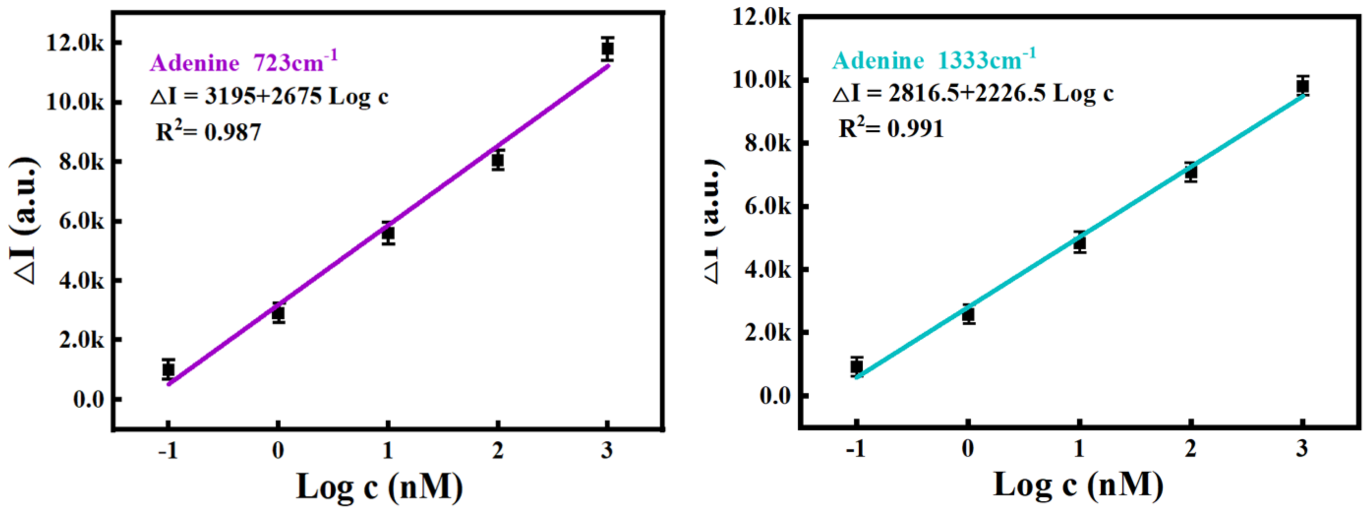

Supplementary Figure S6. Calibration plot of SERS intensity versus logarithm of concentration at 723 cm^{-1} and 1333 cm^{-1} for adenine.

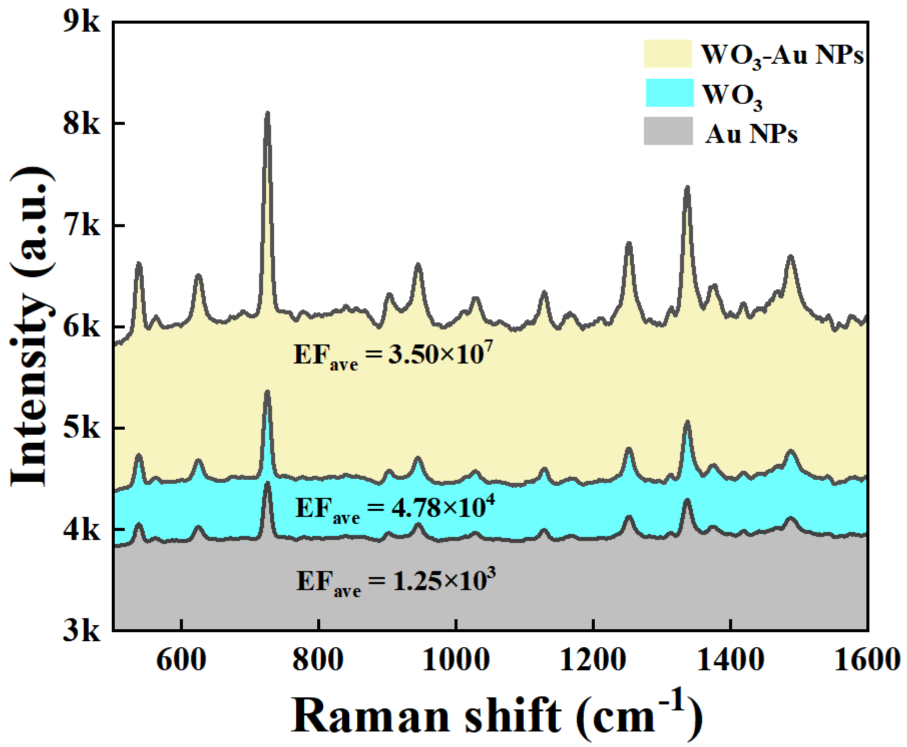

Supplementary Figure S7. Raman spectra (average EFs) of WO_3-Au NPs, WO_3 and Au NPs.

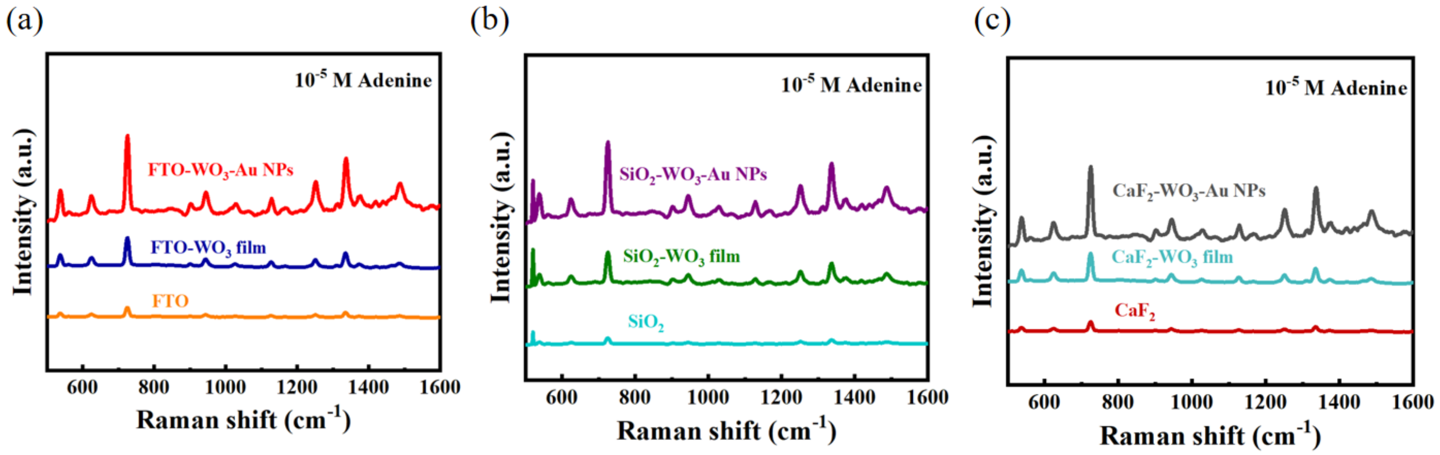

Supplementary Figure S8. (a) Raman spectra of adenine molecules at 10^{-5} M concentration detected by FTO/FTO-WO_3 film/FTO-WO_3-Au NPs. (b) Raman spectra of adenine molecules at 10^{-5} M concentration detected by SiO_2/SiO_2-WO_3 film/SiO_2-WO_3-Au NPs. (c) Raman spectra of adenine molecules at 10^{-5} M concentration detected by CaF_2/CaF_2-WO_3 film/CaF_2-WO_3-Au NPs.
